# The SARS-CoV-2 Entry Inhibition Mechanisms of Serine Protease Inhibitors, OM-85, Heparin and Soluble HS Might Be Linked to HS Attachment Sites

**DOI:** 10.3390/molecules27061947

**Published:** 2022-03-17

**Authors:** Antony Cheudjeu

**Affiliations:** La Colombe Health Center, Fokoue P.O. Box 02, Cameroon; contact@unaffiliated-researchers.com; Tel.: +33-78-569-2507

**Keywords:** COVID-19, D-xylose, camostat mesylate, nafamostat mesylate, alpha-1-antitrypsin, type 2 diabetes, viral infection, serine protease inhibitor, competitive inhibition

## Abstract

This article discusses the importance of D-xylose for fighting viruses (especially SARS-CoV-2) that use core proteins as receptors at the cell surface, by providing additional supporting facts that these viruses probably bind at HS/CS attachment sites (i.e., the hydroxyl groups of Ser/Thr residues of the core proteins intended to receive the D-xylose molecules to initiate the HS/CS chains). Essentially, the additional supporting facts, are: some anterior studies on the binding sites of exogenous heparin and soluble HS on the core proteins, the inhibition of the viral entry by pre-incubation of cells with heparin, and additionally, corroborating studies about the mechanism leading to type 2 diabetes during viral infection. We then discuss the mechanism by which serine protease inhibitors inhibit SARS-CoV-2 entry. The biosynthesis of heparan sulfate (HS), chondroitin sulfate (CS), dermatan sulfate (DS), and heparin (Hep) is initiated not only by D-xylose derived from uridine diphosphate (UDP)-xylose, but also bioactive D-xylose molecules, even in situations where cells were previously treated with GAG inhibitors. This property of D-xylose shown by previous anterior studies helped in the explanation of the mechanism leading to type 2 diabetes during SARS-CoV-2 infection. This explanation is completed here by a preliminary estimation of xyloside GAGs (HS/CS/DS/Hep) in the body, and with other previous studies helping to corroborate the mechanism by which the D-xylose exhibits its antiglycaemic properties and the mechanism leading to type 2 diabetes during SARS-CoV-2 infection. This paper also discusses the confirmatory studies of regarding the correlation between D-xylose and COVID-19 severity.

## 1. Introduction

As of 14 February 2022, just two years after the start of the COVID-19 pandemic, there have been a total of just over 412 million cumulative cases of COVID-19 worldwide, with approximately 5.8 million deaths [1].

Although a race against the clock has allowed developed countries to pool their efforts to implement vaccines and vaccinate a large part of their populations, it remains necessary to find an effective drug against this disease. Indeed, although vaccines have shown their effectiveness against the development of severe forms of the disease [2], they do not completely prevent infection and their effectiveness remains limited over time, especially with the development of new variants [2].

Another major point is the availability of good quality vaccines for low-income countries, particularly sub-Saharan Africa countries, in addition to some countries in Asia and South America [3]. Added to this is the reluctance of most of these populations to be vaccinated, especially in sub-Saharan Africa countries [4].

All this makes it essential to find a drug and ideally a bioactive compound effective against SARS-CoV-2.

Two previous articles [5,6] formulated and discussed why the best strategy for fighting viruses, including SARS-CoV-2, using core proteins as receptors would be the stimulation of HS biosynthesis [5]. We showed that the positions where HS, CS, DS, and Hep chains are attached to core proteins (syndecans and glypicans) are O-glycosides, and that the hydroxyl groups (-OH) of these serine residues (HS binding sites on core proteins) that we call HS attachment sites are the probable binding sites of viruses, including SARS-CoV-2, that use core proteins as receptors [5]. From this hypothesis, we were able to give a first explanation of the occurrence of type 2 diabetes during viral infections [5,6], and the strategy promoting the biosynthesis of GAGs that have D-xylose as the first element of their chains (HS/CS/DS/Hep).

Indeed, if to bind to the core proteins which are their receptors, viruses bind to the position that D-xylose would have occupied to initiate HS biosynthesis; then in the presence of xylose which stimulates the HS biosynthesis, almost all HS attachment sites will be occupied by D-xylose and thus prevent viral attachment.

Although this strategy promoting the biosynthesis of HS is supported by several strong arguments, including the stimulation of HS biosynthesis by heparin, which inhibits almost all these viruses, and the antiglycaemic properties of D-xylose. However, answering the following questions will help to continue corroborating this strategy:

(a) In previous articles [5,6], we hypothesised that viruses prevent the biosynthesis of GAGs (HS/CS/DS/Hep) by attaching themselves to HS attachment sites, which leads to sugars that should be used to make these chains being found in the blood, leading to type 2 diabetes. The question remains as to whether variation in the amount (mass) of GAGs containing D-xylose in the body of a normal healthy person may be sufficient to justify an increase in blood sugar;
(b) Since the previously formulated hypothesis on the binding of viruses, including SARS-CoV-2, at the HS attachment sites on the core proteins, is central, it is important to ascertain whether there are other corroborating facts of this hypothesis in addition to that already provided in the previous article [5];
(c) SARS-CoV-2 has other receptors and cofactors at the cell surface, such as angiotensin-converting enzyme 2 (ACE2) and transmembrane serine protease 2 (TMPRSS2), in addition to syndecans 1 and 4. Therefore, why do we favour the strategy of stimulating the biosynthesis of GAGs (by D-xylose)? In various studies, researchers proposed other strategies—for example, considering using serine protease inhibitors to fight SARS-CoV-2 [7,8,9,10,11];
(d) Ascertain whether there is any link between serine protease inhibitors that inhibit SARS-CoV-2 viral entry and HS attachment sites;
(e) Are there still doubts that bioactive D-xylose molecules initiate GAG biosynthesis? Specifically, can molecules, and not only xylosyltransferase enzymes initiate the biosynthesis of HS chains;
(f) The interpretation of the results of Zheng et al. [12] regarding the correlation between the D-xylose and COVID-19 severity.

To address these various points, we briefly go back to the basic definitions of “competitive inhibition” of serine protease and serine protease inhibitors, and their mechanisms of action.

A preliminary estimation of the mass of sulfated GAGs, except for keratan sulfates, in the body—i.e., the mass of GAGs that share the same linkage region (D-xylose-galactose-galactose) to the core proteins: heparan sulfate (HS), chondroitin sulfate (CS), dermatan sulfate (DS), and heparin (Hep)—is performed through a very brief review of the literature, to support the mechanism by which D-xylose reduces blood sugar. Following this, we corroborate the explanation for the appearance of type 2 diabetes mellitus (T2DM) during viral infection described in previous publications [5,6].

## 2. Binding of Viruses (Including SARS-CoV-2) and HS/CS Attachment Sites on Core Proteins

In a precedent article we identified more than a dozen viruses which use proteoglycans as receptors on the cell surface and are inhibited by heparin: HSV-1, HSV-2, HPV-16, HPV-31, HVB, HVC, HIV-1, HTLV-1, SARS-CoV-2, HCMV, DENV-1, and DENV-2 [5].

Although we previously provided [5] rather lengthy and detailed supporting facts of the hypothesis that these viruses probably bind to serine residues intended to receive the D-xylose molecule for initiating HS chains, we offer other supporting facts to continue to corroborate this hypothesis.

We begin by recalling the two different types of inhibition: competitive and non-competitive.

Non-competitive inhibitors irreversibly bind to different sites, i.e., other than active sites, and induce changes to prevent substrate binding to the active site or so that the enzymes no longer catalyse the reaction [13].

In a situation of competitive inhibition, the exogenous element called the competitive inhibitor binds to the active site and prevents the substrate of the enzyme from binding there. In this case, the rate of inhibition is directly proportional to the concentration of the competitive inhibitor (exogeneous element) [14]. Therefore, logically, precisely knowing the location of the binding site of a substrate is enough to know where its competitive inhibitor binds. We apply this to viruses for which heparin or soluble HS are competitive inhibitors.

Binding sites at the cell surface of soluble unfractionated heparin (UFH) and soluble heparan sulfates have been the subject of several studies since the 1980s.

Wilson et al. [15] in 1990 showed that, in an in vitro study on the heparin/heparan sulfate binding sites at the cell surface of epithelial cells, the sulfate residues (*O*- and *N*-sulfate) of heparin and heparan sulfate played an important role in the recognition of the binding sites of the latter, but were not required. They also showed that endogenous heparan sulfate inhibits heparin binding to the surface of epithelial cells, and that the binding sites of heparin/heparan sulfate were associated with solidly attached proteins to the surface of cells [15].

These results of Wilson et al. [15] indicate that the binding sites of exogenous heparin/heparan sulfate at the surface of epithelial cells were probably the HS/CS attachment sites on the core proteins (i.e., the hydroxyl groups of Ser/Thr residues of the core proteins intended to receive D-xylose molecules to initiate HS/CS chains).

In 2008, Trindale et al. [16] confirmed the conclusions that could be drawn from the study of Wilson et al. [15].

Indeed, in an in vitro study on endothelial cells, Trindale et al. [16] found that the presence of endogenous HS inhibited the binding of heparin (with the same results as those of Wilson et al. on epithelial cells [15]). After degrading the endogenous HS of endothelial cells, heparin was able to bind to the cell surface, and Trindale et al. concluded that the endogenous HS occupied the heparin binding sites at the surface of endothelial cells [16].

Since the endogenous HS are attached to the cell surface at the HS attachment sites on the core protein, the results of Trindale et al. [16] imply that free exogenous heparin/heparan sulfate probably binds at the HS attachment sites on core proteins (syndecans, glypicans). Therefore, what is the direct consequence for the dozens of viruses [5] that utilise core proteins as receptors at the cell surface, and for which soluble heparin and soluble HS are competitive inhibitors, as is the case for SARS-CoV-2 [17,18]?

By definition of “competitive inhibitor”, it appears that there are common binding sites on the core proteins (syndecans in the case of SARS-CoV-2) between these viruses and heparin, and that these binding sites are therefore HS attachments sites, i.e., the hydroxyl groups of Ser/Thr residues of the core proteins intended to receive the D-xylose molecules to initiate the HS/CS chains.

However, surface plasmon resonance (SPR) analysis of the binding of the SARS-CoV-2 Spike protein to immobilised unfractionated heparin and vice versa has been performed.

For instance, it is true that the interaction of viruses with polyanionic HS chains is well-established.

There is a big difference between the binding of heparin and soluble HS on SARS-CoV-2 Spike protein, the binding of the latter on immobilised unfractionated heparin, as is the case in the Claussen et al. study [18], and the binding of these viruses on the cell-layer HSPG.

The first situation (binding of heparin to SARS-CoV-2 Spike protein) seems quite logical, and has been well addressed and discussed in a previous paper, in Section 3 [5].

Concerning the second situation, it is true that different results of the binding of SARS-CoV-2 Spike protein to immobilised unfractionated heparin in SPR analysis have not been discussed in the previous paper. In fact, the studies of Claussen et al. [18] and Tandon et al. [17], for example, showed that the Spike protein of SARS-CoV-2 binds to immobilised unfractionated heparin in the SPR analysis. Additionally, in the conclusion to their studies, Claussen et al. [18] concluded that HS chains on the cell surface promote SARS-CoV-2 infection [18].

However, as we mentioned in our previous article [5], just the fact that UFH significantly and spontaneously stimulates the biosynthesis of cell-layer HS [16,19,20] is in contradiction with Claussen et al.’s conclusion that HS chains promote SARS-CoV-2 infection of various target cells, since UFH inhibits the viral attachment of SARS-CoV-2 in vitro.

Thus, how could we explain the results of the SPR analyses of the binding of SARS-CoV-2 on immobilised UFH or HS chains?

There is a situation where the binding of the SARS-CoV-2 Spike protein on immobilised UFH, as demonstrated in SPR analyses, would not result in the conclusion that the cell-layer HS chains promote infection. This situation occurs if the Spike protein of SARS-CoV-2 attaches specifically to one end of the immobilised UF heparin, the extremity containing xylose, which is the reducing end. Indeed, the hydroxyl group that is attached to the anomeric carbon atom of D-xylose at the extremity of the linkage region (Xyl-Gal-Gal) of the HS/Hep chains is inaccessible for cell-layer HS chains, which are attached to the core proteins at the cell surface. This situation could explain the binding of the virus to immobilised UF heparin without the conclusion being that cell-layer HS promotes infection. For this, xylose should play a major role in the antiviral activity of xylose-containing polysaccharides. 

Studies by Recalde et al. [21] on HSV-1, HSV-2 and DENV-2 viruses, which also use HSPGs as receptors, showed the importance of xylose on the antiviral activity of xylomannans. After determining the structure of two sulfated xylomannans extracted from red alga, they subjected them to Smith degradation to remove the xylose chains. Then they compared antiviral activities, before and after xylose removal, using heparin as a positive control. The antiviral activity of xylose-free polysaccharides obtained after Smith degradation, without alteration of sulfation, was 2.7 to 14 times lower than that of xylomannans [21], thus, indicating the importance of xylose on the antiviral activities of these polysaccharides, as concluded by the authors. Moreover, D-xylose is one of the few bioactive compounds with a strong correlation with the severity of COVID-19, as confirmed by Zheng et al. (see Section 4.3).

Another study, by Yue et al. [22], allowed the authors to conclude that the binding of SARS-CoV-2 on the cell surface was correlated with the expression of cell-layer HS. This was concluded after analysing the binding of SARS-CoV-2 on different cell types, which express different levels of HS on their surface. Without going back to the controversial aspect of the stimulative effect and increase in cell-layer HS by heparin, which inhibits the viral attachment of the same virus on these cells, we can, from the point of view of the hypothesis that we defend here, think that the correlation observed by Yue et al. [22] is an indirect correlation with the number of HS/CS attachment sites. That is to say, this correlation could be indirect with the level of core proteins on the surface of the cells, given the essential character of core proteins for the presence of cell-layer HS.

In addition, in the same study, it was reported that *N*- and *6*-*O*-sulfation are required for the binding of heparin to SARS-CoV-2, but surprisingly that *2*-*, 6*- and *3*-*O*-sulfation were not required for SARS-CoV-2 binding on cell-layer HS [22]. This last point suggests that conclusions should be drawn with caution, after SPR analyses on the binding of SARS-CoV-2 to immobilised heparin. This point goes in the direction of the single situation described above, which would not enter into controversy with the increase in cell-layer HS by heparin. Since, as summarised in Table 1, Wilson et al. [15] have shown that *N*- and *O*-sulfation are important for the recognition of binding sites, and in the context of the situation described above, these binding sites would be inaccessible for cell-layer HS. This is in line with Hudak et al.’s [23] results, where Hudak et al. showed that SARS-CoV-2 binds well to both the cell binding domain (CBD) of syndecan-4 without any HS chains and the region with HS chains and HS attachment sites. These results have been discussed previously [5].

The mechanism by which D-xylose has its antiglycaemic properties, discussed in Section 4 of this article, also supports the hypothesis defended here, since the appearance of T2DM becomes a logical consequence of viral attachment.

In all cases, additional studies will be necessary to fully elucidate what may appear a priori as a controverse to studies on viruses, heparin and HSPGs.

## 3. Other Receptors or Cofactors of SARS-CoV-2: ACE2/SDC1-4/ADAM17/TMPRSS2

SARS-CoV-2, as with many other viruses (HVC, HIV-1), has several coreceptors or cofactors on the cell surface to bind and penetrate the cell. Peng et al. [25] listed about fifteen molecules composed of receptors, coreceptors, and cofactors involved in the binding and entry of SARS-CoV-2 into cells [25]. In Table 2 below, the essential or non-essential characteristics of a few of these are summarised.

We previously addressed the fact that a virus with glycoproteins on its envelope having several receptors, coreceptors, or cofactors, is quite normal [5]. Therefore, we now focus on the receptors or cofactors whose inhibition also causes the inhibition of SARS-CoV-2.

Indeed, the SARS-CoV-2 receptor that is the subject of most studies is the angio-tensin-converting enzyme 2 (ACE2), and yet several studies examining the impact of its inhibition showed that the effectiveness of inhibiting ACE2 as a therapeutic option for COVID-19 patients seems questionable [37,38].

We previously summarised [5] some studies showing that heparan sulfate proteo-glycans (HSPG) were essential and required for the entry of SARS-CoV-2 into the cell (see also Table 2). We did not discuss studies that proposed an alternative strategy us-ing serine protease inhibitors, nor studies that did not mention HSPG. Indeed, one of the most studied cofactors involved in the entry of SARS-CoV-2 is transmembrane ser-ine protease 2 (TMPRSS2). Several in vitro studies showed that its inhibitors partially block the viral entry of SARS-CoV-2 in vitro, such as camostat mesylate [7,9], nafa-mostat [8,9], and alpha-1-antitrypsin [10,11].

These studies with serine protease inhibitors, which do not mention HSPG, cast doubt on the essential character of HSPG on viral entry. A crucial question arises here: would it not be contradictory if the same virus that has independent receptors or co-factors, but that inhibits one of the receptors or cofactors alone, inhibited viral entry?

We discuss why the serine protease inhibitors mentioned above that inhibit the entry of SARS-CoV-2 might have a link with HS attachment sites on core proteins. We now revisit the basic definitions associated with serine protease.

Serine proteases are enzymes that cleave peptide bonds of proteins that have serine residue as the nucleophilic amino acid at their active site. The active-site nucleophile is the hydroxyl group (-OH) of the serine residue attaching the carbonyl carbon of the peptide of the substrate. Blocking this -OH group therefore inhibits the functions of the enzyme (serine protease) [39]. There are several families of serine protease, for example, the trypsins, chymotrypsins, and subtilisins [39,40].

The transmembrane serine protease 2 (TMPRSS2) is part of the chymotrypsin family. The active site of the serine proteases in this family is located at a single position, serine 195 (Ser-195) [39]. Thus, the serine protease inhibitors (substrate for inhibition) of TMPRSS2, namely, camostat mesylate, nafamostat, and alpha-1 proteinase inhibitor, bind to the hydroxyl group of Ser-195 of TMPRSS2 to inhibit it. This is explained well in the mechanism of action of alpha-1 proteinase inhibitor (DrugBank accession num-ber DB00058).

Thus, given the characteristics of their binding sites and their mechanism of ac-tion, these serine protease inhibitors can be suspected of being HS attachment site in-hibitors, by also binding to the hydroxyl group of serine residues of core proteins in-tended to receive the D-xylose for initiating the HS chains. Anyway, the patent WO2013166163A1 from Duke University [41] shows that all three of these serine pro-tease inhibitors and many others are HS serum inhibitors—they inhibit the activity of elastase, which is a protease that sheds the HSPGs at the cell surface. We recently dis-cussed why the entry into cells by endocytosis of viruses that use core proteins as re-ceptors might be a direct consequence of the endocytosis entry of proteoglycans during their renewable process after their shedding from the cell surface [5]. Thus, inhibiting the elastase activity, as serine protease inhibitors do, reduces the endocytosis (See Section 5) entry into cells by SARS-CoV-2 attached at the HS/CS attachment sites, as seen in Section 2 or anywhere on the HSPGs complex.

Guéant et al. [42] found that the blood neutrophil elastase was dramatically in-creased in COVID-19 patients compared with the controls. Additionally, they pro-posed that the latter can be used as a biomarker for COVID-19 severity [42].

It is important to note that, in general, after their shedding from the cell surface, around 30% of syndecans are released into the surrounding extracellular matrix and 70% of all syndecans shed from the cell surface are endocytosed as part of their renew-able process (Section 5).

The ectodomains of syndecans, which are the receptors of SARS-CoV-2, are also shed from the cell surface by a family of enzymes called A disintegrin and metalloprotease (ADAM). ADAM17 is a metalloprotease, the inhibition of which prevents the shedding of syndecan-1 and -4 [43]. We recently discussed why the entry into cells by the endocytosis of viruses that use core proteins as receptors could be the direct conse-quence of the renewable process of proteoglycans after their shedding from the cell surface [5]. We also summarised studies that showed that HS chains maintained on the core protein inhibit metalloproteinase enzymes and thus inhibit their shedding from the cell surface, which thereby reduces the endocytosis (See Section 5) entry of viruses attached to core proteins [5]. In consequence, ADAM17 inhibition could have protective effects on COVID-19 patients [32,33].

## 4. COVID-19 Severity, D-xylose and Type 2 Diabetes: Mechanism by Which the D-xylose Lowers the Blood Sugar and Correlation of D-xylose with COVID-19 Severity

### 4.1. Bioactive D-xylose Molecules: Biosynthesis of HS/CS/DS Chains, Antiglycaemic Properties

Several previous studies showed that exogenous D-xylose has antiglycaemic properties [44,45,46] and acts as a complementary molecule in the regulation of blood sugar [47,48]. The underlying mechanism of the blood sugar reducing function of D-xylose might be related to the action of D-xylose on insulin.

Several studies have investigated the action of D-xylose on insulin and vice versa.

For the actions of insulin on D-xylose, insulin increases the rate of D-xylose penetration into cells two- to five-fold, and at equilibrium, xylose is present in 80% instead of 50% to 55% in cytosol, and 20% in plasma [49].

Insulin also accelerates the rate of D-xylose removal from blood (only in nondiabetic patients; no effect on D-xylose blood level was shown in insulin-treated diabetic patients) [50].

Concerning the effects of D-xylose on insulin, Kim et al. [47] performed an in vivo study on rats, and reported that D-xylose, in addition to significantly reducing the level of sugar in the blood, induces a slight secretion of insulin by β-cells in diabetic rats [47]. This result could suggest that it is through this slight secretion of insulin that D-xylose supplementation reduces the level of sugar in the blood. However, this does not seem to be the case, as Kim et al. [47] also noted that there was no significant change in insulin levels in all groups of diabetic rats. In addition, Jun et al. [51] noticed in an in vivo study on 50 Korean individuals that the administration of D-xylose significantly lowered the serum levels of insulin after 15 and 30 min. This, although not in line with all the observations of Kim et al. [47], leads to the conclusion that the antiglycaemic properties of D-xylose are due to a mechanism other than that of insulin. The results from Bae et al. [48] in 2011 also show a significant decrease in insulin levels after D-xylose consumption. Vanis et al. [52] also showed in a human clinical trial that xylose had a minimal effect on insulin in the serum.

The mechanism by which D-xylose reduces the level of blood seems to be the stimulation of the biosynthesis of GAGs, as we previously formulated [6].

It is important to highlight here this primordial aspect of D-xylose on GAGs, and to specify that the biosynthesis of HS/CS/DS and Hep is initiated by, not only D-xylose derived from uridine diphosphate (UDP)-xylose (substrate of xylosyltransferase enzymes [53]), but also bioactive D-xylose molecules (including exogenous D-xylose) [54,55,56,57,58]. This remains true even in the situation where cells were previously treated with GAG inhibitors [55].

**Figure 1:**
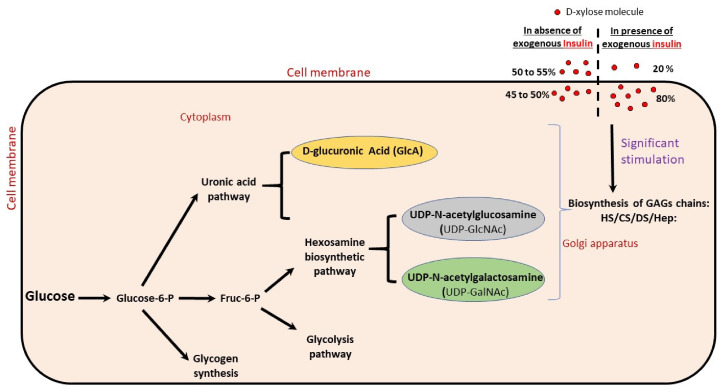
Distribution or utilisation of glucose metabolites in the cell.

To corroborate that it is by the stimulation of HS/CS biosynthesis that D-xylose lowers the level of blood sugar, we conducted a preliminary estimation in Section 4.2 of the minimal amount (mass) of xyloside GAGs in the body of a normal person, which we put into perspective with the amount of glucose/glucagon in the body without ignoring the glycaemic index of the different sugars that compose the chains of these GAGs, especially since these sugars are direct metabolites of glucose (see Figure 1).

Insulin transports more D-xylose in cells; in noninsulin conditions, 50% of D-xylose is both in the plasma and inside the cell, while insulin shifts these proportions to 20% in the plasma and 80% in the cell (see Figure 1) [49]. Free D-xylose molecules significantly stimulate the biosynthesis of HS, CS, and DS [54,55,56,57,58]. Thus, insulin stimulates the biosynthesis of cell-layer GAGs, since it helps transport more D-xylose into the cell, which then stimulates the biosynthesis of GAGs, as confirmed by a previous study [59]. In addition, in 2009, Han et al. [60], in a study on changes in HS of endothelial cells in diabetes, showed that high glucose or insulin levels alone reduced endothelial GAGs, but the presence of both simultaneously, i.e., hyperglycaemia + insulin, preserved GAGs [60]. When both insulin and glucose levels are high, what happens to D-xylose? At high concentrations of insulin (in normal glucose levels), 80% of xylose is able to pass through the cell membrane, but this transport is competitively inhibited by glucose in situations of hyperglycaemia [61]. Thus, high insulin and glucose levels lead to a situation (level of D-xylose in the plasma) comparable to normal conditions without diabetes, as was demonstrated in vivo by Field and Johnson in 1960 [50], who reported the removal of D-xylose from the blood in nondiabetic patients, and no effect on the blood level of D-xylose in insulin-treated diabetic patients. The results from Han et al. [60] in 2009 are thus in line with the action of insulin on D-xylose.

These various studies suggest that the biosynthesis of GAGs is of particular interest for blood sugar control.

### 4.2. Deregulation of GAGs during Type 2 Diabetes: Estimation of Minimal Mass Variation of HS/CS/DS and Hep

The deregulation of GAGs in different pathologies has been frequently observed. In Section 5 of [6], we summarised studies that showed that the biosynthesis of HS/CS/DS was reduced by approximately 14% in T2DM, characterised by a reduction in the activity of xylosyltransferase enzymes.

Additionally, in 1995, Cechowska-Pasko et al. [62], in an in vivo study on diabetic rats, showed that the level of GAGs significantly decreased (by 50% to 70%) in the skin of the rats. In 2021, Dhounchak et al. [63] showed that β-cell failure in T2DM results from HS/HSPGs deficiency; this paper complements previous articles that discussed the importance of preserving HS during inflammatory processes and viral infections [5,6].

In addition to these positive properties, GAG chains are sugar reservoirs of glucose metabolites. Our preliminary estimation of the mass of sulfated GAGs that have D-xylose as the core protein binding element, including HS/CS/DS and Hep (Table 3), suggests that these masses are not negligible (compared with liver glycogen storage capacity).

The minimal and very conservative mass of HS/CS/DS and Hep in the body is approximately 130 g (when taking into account their fractions in different GAGs’ con-centration, as summarised in Table 3).

The proportion of D-xylose in each of the four types of GAGs above (HS, CS, DS, and Hep) is extremely low compared with other sugars such as N-acetylglucosamine and glucuronic acid. This is because D-xylose occupies only the first position in each chain of these different GAGs.

Therefore, if D-xylose is not attached to the core protein, HS, CS, DS, and Hep bio-synthesis cannot take place. Indeed, given the first position of D-xylose, in its absence, the biosynthesis of these GAGs does not initiate, and other (majority) sugars that should be used to form their chains are found in the blood.

In summary, T2DM is accompanied by a fairly significant decrease in GAGs: HS, CS, and DS, up to 50–70% for the skin [62] and approximately 14% overall. A 70% reduction in the levels of HS/CS/DS in the skin alone (see Table 3) is equivalent to the loss of approximately 15 g of GAGs’ chain sugars, while applying 14% to the minimal estimated total of HS, CS, and DS in the body corresponds to approximately 18 g. Putting these very preliminary estimate variations (15–18 g) into perspective with liver storage capacity or with the total amount of glucose in the blood lends credence to the hypothesis that the stimulation of the biosynthesis of GAGs containing D-xylose (HS/CS/DS) as a mechanism explains its antiglycaemic properties.

### 4.3. Correlation of D-xylose with COVID-19 Severity

Zheng et al. [12] reported that there was a correlation between D-xylose levels and the severity of COVID-19 in female patients, corroborating our prediction [6]. Of a total of around 2700 identified metabolite peaks in the plasma of non-severe COVID-19 patients, Zheng et al. [12] selected and analysed around 2000 metabolites. After a second selection of important metabolites, they evaluated the relationship between metabolic changes and clinical outcomes through Spearman’s correlation analysis. The results of this analysis selected approximately five metabolites, including D-xylose (in female patients), which have a very strong (based on the colour scale) correlation with parameters of lung function [12].

On the basis of the reported results about reduced D-xylose level in the plasma of female non-severe COVID-19 patients after discharge, Zheng et al. [12] concluded that the suggestion to use D-xylose as a possible therapeutic drug for COVID-19 was not supported by the data. Unfortunately, it seems that their interpretation of their data on D-xylose was misjudged. In fact, their results concerning D-xylose actually support its therapeutic use for COVID-19. Indeed, both D-xylose-derived UDP xylose and bioactive molecules of D-xylose initiate the biosynthesis and secretion of GAGs [54,55,56,57,58].

Thus, for a given (invariable) amount of bioactive D-xylose in the body (plasma and cytosols), by default, the amount of D-xylose in the plasma is strongly and negatively linearly correlated with the amount of D-xylose inside the cells, and this with the degree of HS/CS/DS biosynthesis. However, Schmidt et al. proved that countering HS degradation inhibits lung inflammation and lung injury [79]. Thus, a reduced D-xylose level in the plasma of COVID-19 patients after discharge (less inflammation) seems to be logical.

Therefore, if viruses and D-xylose bind at the same places (HS or CS attachment sites on core proteins), as we defended in Section 2 and recently [5], it will be logical to administer D-xylose to increase the chances (proportion) that D-xylose molecules bind to the core protein (to initiate HS/CS/DS chains) before the viruses, with which the latter compete. This is a competition based on the proportion of D-xylose and viral load.

## 5. Discussion and Conclusions

In Section 2, we provided additional supporting facts that viruses that use core proteins (syndecans, glypicans) as receptors at the cell surface (as is the case for SARS-CoV-2 [23]) probably use HS attachment sites on the core protein as binding sites. These sites are the hydroxyl groups of serine or threonine residues intended to receive the D-xylose molecule for initiating HS, CS, DS, and Hep chains, providing additional support to the previous article [5].

If the virus, by means of its binding sites on the core protein, prevents the biosynthesis of chains of HS, CS, DS, and Hep (by occupying the place of D-xylose), the unused sugars will be found in the blood, explaining the increase in the level of sugar in the blood during these viral infections (Section 4). A preliminary estimation of the mass variation of these chains in type 2 diabetes (Section 4.2) helps us to corroborate this explanation, especially since the bioactive D-xylose molecule stimulates the biosynthesis of GAGs and has antiglycaemic properties (Section 4).

To inhibit the binding of viruses, such as SARS-CoV-2, on the hydroxyl group of the serine or threonine residues of core proteins, several options are available, some of which related to HS are summarised below:

1. Completely inhibit the biosynthesis of proteoglycans (core proteins and glycosaminoglycans) or keep core proteins and inhibit only GAGs’ chains by heparinase; we discussed the link between the inhibitory properties of heparinase and HS biosynthesis [5]. These options induce the deregulation of endothelial glycocalyx and promote inflammation [79];
2. Use heparin as a competitive inhibitor: heparin not only binds at HS attachment sites, but also stimulates HS biosynthesis; thus, in both cases, it induces occupation of the potential binding sites of viruses (Section 2) [5];
3. Use soluble HS as competitive inhibitor: soluble HS probably binds at HS attachment sites (Section 2), thus preventing the binding of SARS-CoV-2. In the in vitro studies of Tandon et al. [17], all xyloside GAGs (heparin, HS, DS, chondroitin sulfates D, and E) were able to compete with SARS-CoV-2 for its binding to immobilised heparin [17]. The soluble keratan sulfates that are the unique sulfated GAGs without D-xylose (at first position) were also tested in this study and failed to compete with SARS-CoV-2 [17];
4. Serine protease inhibitors, such as camostat mesylate, nafamostat mesylate, and alpha-1-antitrypsin, in addition to inhibiting TMPRSS2, also inhibit the elastase activity, thus reducing the shedding of syndecans and then the endocytosis entry of SARS-CoV-2; in addition, they potentially bind at HS attachment sites (Section 3);
5. Inhibit metalloproteinase enzymes (ADAM17) that shed core proteins and induce virus endocytosis [5]. The biosynthesis of HS inhibits metalloproteinase enzymes (Section 3);
6. Use a bioactive compound as a competitive inhibitor: D-xylose, for example, stimulates the biosynthesis of xyloside GAGs (HS, CS, DS) (Section 4), thus preventing the binding of SARS-CoV-2. Xylitol was also shown in several studies to be a competitive inhibitor of SARS-CoV-2 [80]. D-xylose/xylitol has many other properties in relation to the stimulation of HS biosynthesis [6];
7. Another option recently studied in an in vivo study by Fang et al. [81] is the use of bacterial lysate OM-85 Broncho-Vaxom^®^ for the control of COVID-19. OM-85 is a mixture of *H. influenzae*, *S. pneumoniae*, *K. pneumoniae*, *Klebsiella ozaenae*, *S. aureus*, *Streptococcus pyogenes*, *Streptococcus viridans*, and *M. catarrhalis* [82]. Among them, at least one uses syndecan-1 as a receptor at the cell surface; this is the case of *Streptococcus pneumoniae* [83]. As previously stated [5], this would mean that *Streptococcus pneumoniae* binds at HS attachment sites on syndecan-1 (the same potential place as SARS-CoV-2). Heparin, heparan sulfate, and chondroitin sulfate are competitive inhibitors of *Streptococcus pneumoniae* [84]. From Section 2, because heparin and HS are its competitive inhibitors, we deduce once again the probable binding site of *S. pneumoniae* on syndecan-1. Therefore, OM-85 is a competitive inhibitor of SARS-CoV-2, as demonstrated by the results of Fang et al. [81]. However, by preventing HS biosynthesis (through one of its binding sites), bacterial lysate OM-85 causes HS inhibition, heparanase overexpression, and the overexpression of metalloproteinase enzymes (including ADAM17), thus inducing the shedding of the ectodomain of syndecans [5]. All these facts were reported by Fang et al. [81]. In addition, the shedding of syndecan-1 ectodomain by *Streptococcus pneumoniae* was reported [85].

Table 3 shows that the largest contributor by far to the mass of GAGs having D-xylose as a binding element on core proteins is the cartilage, with about 70 g of chondroitin sulfate. What is the impact of COVID-19 or type 2 diabetes on cartilage? Studies reported the inflammation of arthritis in the pathobiology of COVID-19 [86]. However, in 1987, Kleesiek et al. [87], in an in vivo study, showed that the serum of xylosyltransferase, which catalyses the transfer of D-xylose from UDP-xylose to the hydroxyl group of serine residues of core proteins, was a biomarker of cartilage destruction in chronic joint disease [87]. This reflects the importance of CS, the only xylose GAG present in cartilage, for the protection of cartilage. Indeed, the CS of chondrocytes has protective effects by regulating collagen type II synthesis and hyaluronic acid, inhibiting cellular death, and increasing PG production [88,89]. Thus, it protects the chondrocyte glycocalyx and reduces inflammation. The anti-inflammatory properties of CS of chondrocytes were demonstrated well [90,91].

Concerning the importance of the inhibition of proteoglycans shedding, Yanagishita showed in 1992 that 70% of transmembrane proteoglycans (syndecans) shed from the cell surface are endocytosed and 30% are released in the extracellular matrix [92], whereas 100% of glycosylphosphatidylinositol-anchored proteoglycans (Glypicans) are endocytosed [92]. This supports the argument that HSPGs shedding enhances viral endocytosis (Section 3).

It would not be reasonable if some reported facts which seem to be seriously questioning the conclusion that “HS promotes infections” are just ignored. Whether HS biosynthesis stimulation effect and virus entry inhibition of heparin are independent effects or not, does not change the reported facts:

(a) Heparin is a competitive entry inhibitor of dozens of viruses (including SARS-CoV-2) [5];
(b) Heparin significantly and spontaneously stimulates the biosynthesis of cell-layer HS chains (with the same sulfation as highlighted by Nader et al.) (Section 2);
(c) The interaction between HS chains and diverse viruses (including SARS-CoV-2).

If based on fact (c), we conclude that “HS chains promotes infections”, how could we justify that the same heparin which is an entry competitive inhibitor of SARS-CoV-2 spontaneously stimulates by 2- to 3-fold the biosynthesis of the cell-layer HS (infection promoter element)? Especially when the pre-incubation of cells with heparin before infection also inhibits the viral attachment of SARS-CoV-2 [93,94]. The pre-incubation of cells with heparin 30 min before infection, as was the case in the Partridge et al. [93] experiment, significantly increases the amount of cell-layer HS (with the same sulfation), and yet viral attachment is inhibited with this pre-treatment, in addition to studies on the effects of desulfation discussed in Section 2.

Thus, extrapolation of results from the different SPR analyses with immobilised heparin to the cell-layer HS need to be conducted with caution, since there is at least one situation where the three facts listed above could be verified without arriving to the conclusion that “HS promotes infection” (Section 2).

**Figure 2:**
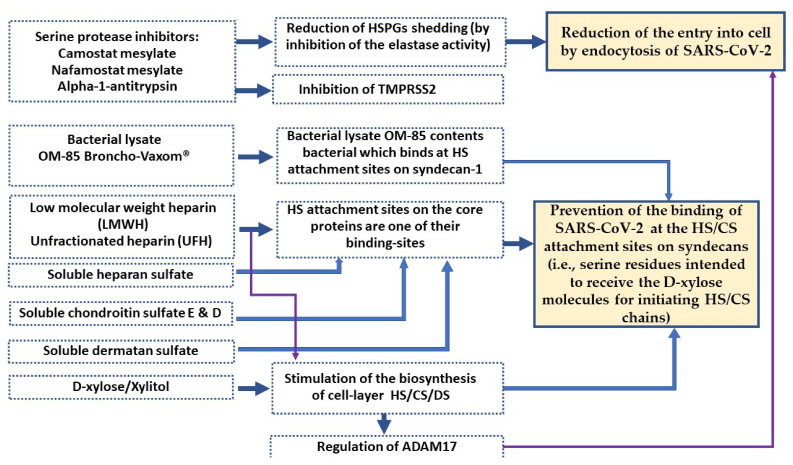
Potential mechanisms of action of each inhibitor during COVID-19.

In summary, there are many indications that the stimulation of HS, CS, and DS biosynthesis appears to be one of the most appropriate strategies of the different options summarised in Figure 2, because in addition to inhibiting viral entry, this strategy reduces inflammation and, depending on the compound used, fights type 2 diabetes induced by viral infection. In addition, Dhounchak et al. [63] showed that β-cell failure in T2DM results from a deficiency in HS/ HSPGs.

To stimulate the biosynthesis of GAGs, D-xylose seems to be the best option, compared with heparin and other xylosides, for already mentioned reasons [5], and the confirmed correlation of D-xylose with the severity of COVID-19 with this assumption (Section 4.3). Indeed, one of the advantages of D-xylose compared with heparin is the recognition of their binding sites. Wilson et al. [15] showed that the sulfation (*O*- and *N*-sulfate) of heparin and heparan sulfate is important in the recognition of their binding sites on the core proteins. Unlike heparin, the bioactive molecule of D-xylose does not need sulfation to attach itself to the position intended for it on the core proteins to initiate the chains of HS, CS, and DS.

## 6. Limitations

We did not discuss the impact of options (OM-85, serine protease inhibitors) that inhibit HS on inflammation. Several other cofactors and potential receptors of SARS-CoV-2 and other competitive inhibitors were not addressed in this article. Another limitation is the non-discussion of other possibilities offered by the inhibition of HSPGs shedding by serine protease inhibitors, as, for example, the fact that inhibiting HSPG shedding may reduce availability of non-cell-bound HS chains that are known to act as cofactors for receptor binding (such as TLR in the WO2013166163A1)

## Figures and Tables

**Table 1:** Binding sites of heparin on the cell’s surface in the absence, and in the presence, of SARS-CoV-2.

	Situation 1 Binding of Heparin to the Cell Surface In the Absence of Any Virus	Situation 2 Binding of Heparin to the cell Surface In the Presence of the SARS-CoV-2
Binding sites on the core protein	Heparin has many binding sites on the cell surface [15]. Wilson and colleagues revealed that some of these binding sites of heparin were on proteins firmly attached to the cell-surface [15]. The results of Trindale and colleagues left them to suggest that these binding sites were the HS attachment sites on the core protein. In fact, Trindale and colleagues concluded that the endogenous HS occupied the heparin binding sites on the surface of endothelial cells [16]. Indeed, in their experiment, heparin was able to bind to the endothelial cell surface, only after the degradation of endogenous HS chains.	Heparin is a competitive inhibitor of SARS-CoV-2 in HEK293T cells [17]. By definition of “competitive inhibitor”, both heparin and SARS-CoV-2 share at least one binding site in common on the HSPGs. Based on Trindale et al. [16], and Wilson et al. [15], these sites seem to be the HS/CS attachment sites on the core protein.
Importance of *N*- and *O*- sulfation in the recognition of binding sites	Wilson et al. [15] showed in 1990 that chemically modified derivatives of heparin, in which either *N*- or *O*- sulfation had been suppressed, markedly reduced the ability of the latter to compete for heparin binding sites on the cell surface.	Tandon and colleagues [17] confirmed that *N*-desulfation of heparin and also *O*-desulfation reduced the ability of heparin to compete with SARS-CoV-2 binding. This appears as a possible direct consequence of the Wilson et al. [15] observation. In fact, as discussed above, heparin shares a common binding site on the core protein with SARS-CoV-2, and *N*- or *O*- desulfation markedly reduced the ability of the heparin to recognise that binding site on the cell surface, according to the Wilson et al. results [15]. Thus, it is not specific to SARS-CoV-2.
Influence of carboxylation in the recognition of binding sites	Wilson et al. [15] also found that the reduction in the carboxyl groups of heparin significantly decreased the ability of the latter to compete for heparin binding sites on the cell surface.	Studies have shown that the SARS-CoV-2–heparin interaction is chain-length dependent [24].
Impact on the cell-layer HS	Heparin significantly spontaneously stimulates the biosynthesis of cell-layer HS [16,19,20]. This stimulation is independent of its binding to the HS attachment sites, as observed by Trindale et al. [16].	The fact that heparin significantly and spontaneously stimulates the biosynthesis of cell-layer HS [16,19,20] does not support Claussen et al.’s conclusion that HS chains promote SARS-CoV-2 infection of various target cells, since heparin inhibits the viral attachment of SARS-CoV-2 in vitro, despite any explanations we would propose, so as not to link the two effects of heparin.
Competition for the binding sites with other xyloside GAGs (HS/CS/DS)	Heparan sulfate and dermatan sulfate are heparin binding competitive inhibitors at the cell surface [15].	All xyloside GAGs (heparin, HS, DS, chondroitin sulfates D, and E) were able to compete with SARS-CoV-2 for its binding to immobilised heparin [17]. The soluble keratan sulfates that are the unique sulfated GAGs without D-xylose at first position were also tested and failed to compete with SARS-CoV-2 [17].

**Table 2:** SARS-CoV-2 entry: essential and non-essential characteristics of some receptors/cofactors.

SARS-CoV-2 Receptors/Cofactors	References	Few Inhibitors of Receptors/Cofactors	Essential Character
HSPGs (Syndecans -4)	[23]	Heparin is used as a competitive inhibitor of the binding of diverse viruses to HSPGS [5]	Inhibition of HSPGs inhibits the viral entry [23,26]. HSPGs are essential for SARS-CoV-2 entry [23,26,27].
ACE2	[7]	DX600 [23,28]	DX600 modestly inhibits SARS-CoV-2 entry [26]. ACE2 is not essential for SARS-CoV-2 entry [26,29].
TMPRSS2	[7,30]	Camostat mesylate [7,9], nafamostat [8,9], alpha-1-antitrypsin [10,11]	Reduction in SARS-CoV-2 entry. TMPRSS2 is not essential for SARS-CoV-2 entry [30]. SARS-CoV-2 binds to cells lacking TMPRSS2 [30].
ADAM17	[31]	Apratastat and TMI-1 [32], TNF-α protease inhibitor 1 (TAPI-1) [33]	ADAM17 inhibitions exert protective effects [32]. ADAM17 is probably not essential for SARS-CoV-2 entry, based on results obtained on SARS-CoV [34].
Cathepsin	[35]	Cathepsin inhibitor (E64D) [35]	SARS-CoV-2 is not inhibited by E64D [35]. Cathepsin is not essential for SARS-CoV-2 entry [30,35].
integrin	[36]	Integrin inhibitor Cilengitide [36]	Cilengitide significantly inhibits SARS-CoV-2 internalisation [36]; the essential or non-essential character of integrin activation for SARS-CoV-2 entry is not unanimous [36].

**Table 3:** Minimal GAG mass estimation (in g) in a healthy person weighing 65 kg.

Elements	GAG Concentration	Estimated Mass of the Element	Estimated GAG Mass (g)
Liver	198 µg/g [64] (HS/CS/DS represent 82%)	Average, 1561 g [65]	0.31
Pancreas	HA (29 µg/g) + HS (176 µg/g) + DS (77 µg/g) + CS (38 µg/g) = 0.32 mg/g [66] (thus HS/CS/DS represent 91%)	Average, 87 g [67]	0.03
Cartilage	149 µg/mg [68] (CS 50%/KS 50%)	Weight of all cartilage in adult humans is estimated to be 1.5% of total body mass [69]	145.3
Lungs	5 mg/g, average of HS/CS/DS [70]	Average, 800 g for the two lungs [65]	4.48
Skin	2.02 mg/g (DS 94%, HS 3%, heparin 3%, and Ch-4S/Ch-6S < 2%) [71]	16% of body weight [72]	21.01
Human cornea tissues	Approximately 115 mg/g [73]	Estimated: 6% of eye weight	0.10
Plasma	Less than 0.5% of total plasma proteins [74]	Plasma proteins constitute approximately 0.5% of total body mass [75] (325 g)	1.63
Blood vessel walls (Venous and arterial walls)	32 mg/g [76] for arteries and approximately 2 mg/g for veins [77]. We used (32 × (12/72) + 2 × (60/72)) mg/g and obtained 7 mg/g as the average. HS/CS (CSB and C6S)/DS represent 96% ([76])	Relative volume of veins is 60% of total blood volume, 12% for arteries [78]	32.76
		Minimal estimated GAG mass (g) in one person (65 kg)	205.62

## Data Availability

Not applicable.
